# Single-Photon Emission Computed Tomography and Positron Emission Tomography Studies of Antisocial Personality Disorder and Aggression: a Targeted Review

**DOI:** 10.1007/s11920-019-1011-6

**Published:** 2019-03-09

**Authors:** Nathan J. Kolla, Sylvain Houle

**Affiliations:** 1 Research Imaging Centre, Centre for Addiction and Mental Health (CAMH), Toronto, Canada; 2 Violence Prevention Neurobiological Research Unit, CAMH, Toronto, Canada; 3 Psychiatry, Criminology, and Psychological Clinical Science, University of Toronto, Toronto, Canada; 4 Research and Academics, Waypoint Centre for Mental Health Care, Penetanguishene, Canada; 5 Research Imaging Centre, CAMH, Toronto, Canada

**Keywords:** Antisocial personality disorder, Positron emission tomography, Single-photon emission computed tomography, Aggression

## Abstract

**Purpose of Review:**

This paper aims to provide a comprehensive discussion of single-photon emission computed tomography (SPECT) and positron emission tomography (PET) studies of antisocial personality disorder (ASPD) and aggression.

**Recent Findings:**

Among ASPD males with high impulsivity, the density of brainstem serotonin (5-HT) transporters shows a relationship with impulsivity, aggression, and ratings of childhood trauma. 5-HT_1B_ receptor (R) binding in the striatum, anterior cingulate cortex, and orbitofrontal cortex (OFC) correlated with anger, aggression, and psychopathic traits in another study of violent offenders, most of whom were diagnosed with ASPD. Finally, the density of monoamine oxidase-A (MAO-A), a mitochondrial enzyme that degrades 5-HT, norepinephrine, and dopamine (DA), was reported as lower in the OFC and ventral striatum of ASPD. Among non-clinical populations, 5-HT_4_R binding, as an index of low cerebral 5-HT levels, has been associated with high trait aggression, but only in males. Furthermore, evidence suggests that individuals with high-activity MAO-A genetic variants compared with low-activity MAO-A allelic variants release more DA in the ventral caudate and putamen when exposed to violent imagery.

**Summary:**

There are very few PET or SPECT studies that exclusively sample individuals with ASPD. However, among ASPD samples, there is evidence of regional serotonergic abnormalities in the brain and alteration of neural MAO-A levels. Future studies should consider employing additional molecular probes that could target alternative neurotransmitter systems to investigate ASPD. Furthermore, examining different typologies of aggression in clinical and non-clinical populations using SPECT/PET is another important area to pursue and could shed light on the neurochemical origins of these traits in ASPD.

## Introduction

Most violent crime is perpetrated by a small group of males who display conduct-disordered behavior in childhood and meet diagnostic criteria for antisocial personality disorder (ASPD) as adults. Although ASPD is not as prevalent as other psychiatric conditions in the community (some estimates indicate that ASPD affects 1% of American adults [1]), nearly 50% of incarcerated individuals meet criteria for the disorder [2]. Furthermore, 85% of individuals with ASPD have acted violently toward others [3, 4]. Hence, these statistics underscore the importance of a research framework that takes into account multiple levels of information to understand the pathology, including neurochemistry, of ASPD.

A common mistake made by clinicians and researchers is the interchangeable use of the terms ASPD and psychopathy. While ASPD and psychopathy share some features in common (e.g., impulsivity and involvement in criminal activity), prototypical psychopathy also includes the personality characteristics of deficient affective experience, lack of empathy, and callousness, which are not necessarily observed in ASPD. Most individuals with psychopathy meet criteria for ASPD, while only 10% of persons with ASPD present with psychopathy [5]. There is also some evidence that psychopathy is a more severe form of ASPD [6].

ASPD is typically diagnosed in clinical and research settings using criteria set forth in the Diagnostic and Statistical Manual of Mental Disorders Version 5 [7]. In contrast, the most common instrument used to classify individuals with psychopathy is the Hare Psychopathy Checklist-Revised (PCL-R) [8]. The PCL-R contains 20 items that are each rated as 0 (not present), 1 (partially present), or 2 (fully present), ideally based on file review and clinical interview. Scores between 0 and 40 are thus generated. In North America, a score of 30 or more denotes the presence of psychopathy. Important to note is that psychopathy is not universally viewed as a taxon (e.g., presence or absence of a disorder) but, instead, is often conceptualized as a dimensional construct [9]. The same is true of ASPD [10].

Aggressive behavior is a common feature of ASPD, psychopathy, and other psychiatric disorders. Healthy individuals without psychiatric illness also engage in aggressive behavior under certain circumstances. As such, trait aggression is thought to occur along a continuum [11], where some people rarely, if ever, act aggressively, while others (usually when psychiatric disorder is present) have lower thresholds for becoming aggressive. Aggression that is impulsive and typically manifests in response to a threat or anger is known as reactive aggression. By contrast, aggression that is premeditated or utilized to achieve an aim or increase dominance is labeled proactive or instrumental aggression [12]. A lack of differentiation of these two main types of aggression often leads to conflicting results in the literature. Research investigating the neural mechanisms of aggressive behavior has also been plagued by this issue, although some positron emission tomography (PET) studies, as discussed below, have differentiated reactive from proactive aggression and report on the neurochemical indices of each. The distinction between reactive and proactive aggression is important, as these entities likely have different neural underpinnings.

The purpose of this paper was to critically examine single-photon emission computed tomography (SPECT) and PET brain imaging studies of ASPD and aggression in non-clinical populations. We did not include psychopathy, given the scarcity of imaging studies that identified participants with this condition. The main search was conducted in PUBMED. An additional search was conducted using the Elton B. Stephens Company (EBSCO) platform that searched the following databases: PsycINFO, PsycArticles, and the Psychology and Behavioral Science Collection. The search of studies involving ASPD participants was limited to articles written in English, but there were no limits set on date of publication. This search was run in PUBMED and EBSCO as follows: (1) (antisocial personality disorder OR borderline personality disorder OR psychopathy OR intermittent explosive disorder) AND (positron emission tomography OR PET OR single-photon emission computed tomography OR SPECT). The search relating to aggression was limited to articles written in English during the past 5 years (2014–2018). Articles that focused on neurodegenerative disorders were not included. The search terms were as follows: (2) (violence OR aggression) AND (positron emission tomography OR PET OR single-photon emission computed tomography OR SPECT). Further studies were identified by examining the references of key articles; additional searching was performed using a citation index. Studies that included at least one subject with ASPD were included in the first analysis.

## SPECT

SPECT is a three-dimensional nuclear imaging technique. Following the intravenous injection of a radiopharmaceutical, a molecule tagged with a gamma emitting radionuclide—most commonly technetium-99 m (^99m^Tc)—a radiation detector rotates around the subject’s body acquiring multiple two-dimensional images. These images are then fed to a tomographic reconstruction program that produces three-dimensional maps of the radiopharmaceutical concentration within the body. The most widely used SPECT radiopharmaceutical for brain imaging is [^99m^Tc]hexamethyl-propylenamine oxide ([^99m^Tc]HMPAO) [13], which provides an index of regional cerebral blood flow (rCBF). Because blood flow in the brain is closely linked to local brain metabolism and energy use, [^99m^Tc] HMPAO can offer an indirect measure of brain metabolism.

## SPECT Studies of ASPD

In the five SPECT studies that follow, 37 individuals with ASPD were scanned out of a total of 198 participants**.** One report that examined rCBF using [^99m^Tc] HMPAO SPECT sampled 40 inpatients hospitalized on an alcoholism inpatient ward [14]. Fifteen of these individuals met criteria for ASPD, 21 had primarily dependent personality disorder, and four had other personality disorders. Axis I comorbidity (besides alcohol misuse) was exclusionary. All individuals were scanned at the termination of alcohol withdrawal symptoms. Ten males who did not drink and had no relatives with alcohol misuse served as the comparison group. The main finding to emerge was that the ASPD group displayed anterior frontal hypoperfusion relative to the other personality groups. A reduction in cerebral lobe rCBF ratio was also reported in 87.5% of the ASPD group. This study is notable for the fact that ASPD participants were tested as a single group rather than part of a heterogeneous sample of aggressive or antisocial individuals.

Another [^99m^Tc] HMPAO investigation [15] that studied healthy participants and detoxified opiate misusers with either depression, ASPD (7 males and 2 females), or no comorbid axis I or II psychopathology found that the ASPD group had lower rCBF in the right frontal lobes compared with healthy control subjects and opiate misusers without comorbid psychiatric conditions. On the other hand, the study reported no differences between the ASPD and depression samples, even though other SPECT studies of depression have reported alterations in several brain regions [16, 17].

A subsequent [^99m^Tc] HMPAO study [18] investigated 32 violent offenders without severe and persistent mental illness; only two subjects had ASPD. There was no comparison group, and statistical tests were limited to correlational analyses. The authors reported significant negative correlations between the interpersonal features of psychopathy (e.g., callousness, lack of empathy, deficient affective experience) and frontal and temporal perfusion. Given the small number of ASPD participants, it would seem that these results have less relevance for understanding cerebral blood flow alterations in ASPD.

A [^99m^Tc] HMPAO protocol [19] scanned 37 participants with either borderline personality disorder (BPD) or ASPD (*n* = 10) and 34 healthy control subjects. Axis I psychopathology was exclusionary for all groups. Compared with the healthy group, reduced rCBF in the right lateral temporal cortex, right frontopolar cortex, and right ventrolateral prefrontal cortex was reported among the participants with personality disorders. Importantly, there was no difference in rCBF between ASPD and BPD, suggesting that these conditions might share similar pathologies, although subsequent PET studies of ASPD and BPD have reported important differences in other neural substrates studied [20, 21••].

An innovative study that utilized [^99m^Tc] HMPAO [22] scanned nine pre-trial violent offenders and then re-scanned them on average 4 years later in what was essentially a test-retest design. Only one participant was diagnosed with ASPD, and there were no healthy controls. Notably, all subjects had improved clinically from the first scan, which included the absence of any psychotic symptoms, drug abuse, or treatment with psychoactive medication. Group results from the first scans were compared with amalgamated data from the second set of SPECT scans. No quantitative differences between groups were detected. Violent offenders demonstrated unchanged frontotemporal hypoactivity from the initial SPECT scan. Moreover, the mean changes in all of the regions sampled were below 5%. These results raise the important question of whether frontotemporal hypoactivity is a state or trait phenomenon in violent offenders and what role, if any, the presence of psychoactive substances contributes to these findings. Of course, since this study included only one ASPD participant, it is difficult to generalize to this population.

Early SPECT studies tended to focus on violent offenders or substance misusers as groups that failed to include large numbers of ASPD participants. As a result, findings may be less relevant to ASPD. These investigations were also likely underpowered to detect differences between patient groups, for example, ASPD versus depression or BPD. There is some evidence to support decreased frontotemporal rCBF in ASPD, but, again, this pattern is observed in offender groups with very little representation from ASPD subjects. However, the phenomenon of frontotemporal hypoperfusion is bolstered by structural magnetic resonance imaging findings in ASPD that document reduced volumes of gray matter in these regions [23–25]. Since the frontal lobes exert control over neural threat response systems, structural or functional alterations of this complex could weaken threat response regulation [26], ultimately leading to increased reactive or impulsive aggression. Finally, some evidence indicates that structural alteration of temporal lobes in ASPD relates to impairment in aggression control, heightened impulsivity, and deficits in emotion processing [27].

## PET

PET is another three-dimensional nuclear imaging technique. It differs from SPECT by using positron-emitting radiopharmaceuticals (PERs) rather than single gamma photon emitting radionuclides. Given the size of the technetium atom and the fact that it must be chelated to the target molecule, the number of useful SPECT radiopharmaceuticals for brain imaging is severely limited. Carbon-11 and fluorine-18 are the most widely used radionuclides for PET. Since the nuclides are nearly identical to the non-radioactive carbon-12 and fluorine-19 atoms, they can radiolabel a much wider range of natural molecules or drugs to probe specific biochemical processes, enzymes, transporter substrates, ligands for receptor systems, hormones, antibodies, peptides, drugs, and oligonucleotides [28]. When positron emitters decay, two annihilation photons are produced, and the PET scanner detects these photons. After intravenous injection in the subject, the PET scanner records the three-dimensional concentration of the PER over time. While PET imaging offers several technical and biological advantages over SPECT, its widespread use is limited by the short half-lives of carbon-11 (20.4 min) and fluorine-18 (110 min), requiring their production by cyclotrons located close to the PET scanners.

Depending on the PER used, different biological indices, such as metabolic rate, binding potential, neuroreceptor occupancy, or distribution volume, can be obtained from the PET data by a mathematical technique known as kinetic modeling. In some instances, simpler measures are used, such as standardized uptake values.

The glucose analog 2-deoxy-2-[fluorine-18]-fluoro-D-glucose ([^18^F]FDG) is the most commonly used PET radiopharmaceutical [29]. Upon injection, [^18^F] FDG is transported into cells and phosphorylated much like glucose to FDG-6-phosphate. However, since FDG-6-phosphate is not a substrate for glucose-6-phosphate isomerase, it remains trapped in the cell, reaching near equilibrium approximately 1 h after injection. For brain scans, the regional cerebral metabolic rate of glucose (rCMRglu) is then measured for individual brain regions.

## FDG PET Studies in ASPD

Among the eight investigations where it was possible to discern the number of ASPD participants who received an FDG PET scan, 36 subjects with ASPD out of 310 participants were scanned. An initial FDG PET study [30] analyzing brain metabolism examined eight patients admitted to a state psychiatric hospital who endorsed repetitive violence and who either had an intermittent explosive disorder (IED), a behavioral condition marked by volatile outbursts of explosion and anger, or ASPD. However, only one individual in the sample met criteria for ASPD. Patients with chronic psychotic conditions were also included in the analysis. Eight healthy controls were additionally tested. Results revealed that violent patients had significantly lower metabolism in the left and right prefrontal regions, left frontal regions, and left and right temporal medial areas. Given the paucity of ASPD participants, it was not possible to test the individual effects of ASPD on the results obtained.

Another FDG PET investigation [31] compared personality disorder subjects (6 ASPD out of 17 total subjects) with 43 healthy controls who had no psychiatric history. The experimental group comprised military personnel admitted to an inpatient ward. No differences in rCMRglu were detected between the ASPD patients and healthy controls. Moreover, there was no interaction between ASPD subjects and the image plane. The authors suggested that a history of alcohol abuse (4 of 6 subjects) may have produced sufficient variability in the data to lead to a false negative finding. Insufficient power was also cited as a possibility. On the other hand, for the six BPD participants, an interaction emerged between diagnosis and image plane. For example, BPD patients showed significant differences between normalized rCMRglu in two planes of the frontal lobes, one showing an increase and the other a decrease. These results tentatively suggest a dissection of neural correlates in ASPD and BPD that further support the notion of these disorders as distinct conditions.

One FDG PET scan examined seven extremely violent offenders [32] (murder was the index offense for all subjects—in all cases unprovoked—and some individuals had multiple victims), one of whom was diagnosed with ASPD. Nine control subjects without organic brain damage were also included. Quantitative PET data were evaluated as standardized uptake values that compared the greatest occipital region with the lowest temporal region. The mean percentage of decreased activity in the violent group was 39% and statistically greater than the mean decrease in the control group (27%). The authors suggested that, similar to other studies, glucose metabolism abnormalities in the temporal lobe were related to violent behavior. As all episodes of violence exclusively involved proactive or instrumental aggression, these results could shed some light on the neural correlates of this form of aggression.

A two-scan FDG PET protocol [33] examined 13 subjects with impulsive aggression (four with comorbid ASPD) and 13 healthy controls. Meta-chlorophenylpiperazine (*m*-CPP), a serotonergic probe, was administered prior to one scan, while the other scan had no stimulus. *m*-CPP is a partial agonist at serotonin (5-HT) 2A and 2C receptors and also targets presynaptic 5-HT sites [34]. PET images were standardized as mean relative glucose metabolic rate (rGMR). The authors reported that, unlike healthy controls, impulsive patients showed no activation in the left anteriomedial orbital cortex in response to *m*-CPP and that the left anterior cingulate cortex (ACC), which is typically activated by *m*-CPP, was deactivated in the experimental group. On the other hand, the posterior cingulate gyrus was activated in impulsive patients and deactivated in healthy controls. No group differences were detected in the baseline scans. The authors interpreted their results to mean that activation of the anterior cingulate gyrus and posterior orbital cortex in concert with serotonergic input may act to constrain aggressive behavior.

Another investigation that employed fenfluramine (FEN), an indirect agonist of 5-HT receptors [35], and FDG PET sampled a cohort of 22 BPD patients, one of whom had comorbid ASPD, and 24 control subjects [36]. Study participants received placebo on day 1 and FEN on day 2 before the PET scans. Gender differences in FDG uptake emerged between BPD participants in terms of response to placebo by diagnosis, within-group comparisons in response to FEN, and between-group comparisons in response to FEN. Importantly, when measures of trait impulsivity and aggression were included as covariates in each of the models, all group comparisons and differences between BPD and control groups in response to placebo were rendered insignificant. These results suggest that impulsive and aggressive personality traits may have been driving the observed associations. Given the small number of individuals with comorbid ASPD, it is not unexpected that none of the analyses controlled for this covariate.

A double-blind, placebo-controlled study of fluoxetine, a selective serotonin reuptake inhibitor (SSRI) was conducted utilizing FDG PET [37]. Subjects received a PET scan before and after 12 weeks of SSRI treatment. Initially, 22 non-depressed, impulsive aggressive patients with BPD (4 subjects had ASPD) were enrolled. However, only 10 of 15 participants on active treatment and three of five taking placebo completed the investigation. The authors did not report how many patients with concomitant ASPD had completed the trial. Results indicated that rGMR was increased in the orbitofrontal cortex (OFC) and medial anterior temporal regions relative to baseline in the SSRI-treated group at the conclusion of the study. The study authors proposed a relationship between increased OFC rGMR and improvement in aggressive symptomatology.

An additional FDG PET study by the same group as above [38] evaluated 38 patients with BPD (10 subjects had comorbid ASPD) and Intermittent Explosive Disorder-Revised (IED-R) and 36 healthy controls in a two-scan protocol employing the Point Subtraction Aggression Paradigm (PSAP). The PSAP is a psychometric task that assesses provoked aggression in a laboratory setting using a computer protocol [39]. Participants’ aggressive responding to the loss of “points” worth money over the 35 min of testing is evaluated. In the study, each participant underwent two PET scans with a provocation and non-provocation version. Patients increased mean (provoked-non-provoked) rGMR in the OFC and amygdala when provoked, while healthy controls showed a decrease in mean rGMR in these same regions. Subgroups involving participants with comorbid major depressive disorder and posttraumatic stress disorder were analyzed (no differences were appreciated), although ASPD as a subgroup was not considered.

A re-analysis of the same dataset [40] revealed that male patients had lower mean striatal rGMR in both provoked and non-provoked groups compared with all other groups. Interestingly, the investigators found no relationship between striatal activity and behavioral aggression among the patients. Also, an ASPD diagnosis was not treated as a covariate. Based on their findings, the study authors opined that the striatum could contribute to social aggression as exemplified by BPD subjects with comorbid IED-R.

Seventy-two adults received an FDG PET scan in a more recent investigation [41] and also completed Cloninger’s Temperament and Character Inventory [42]. Based on these results, 13 subjects (18%) were designated as having an antisocial personality (ASP). None of the participants were administered structured instruments typically used to diagnose ASPD. Among the ASP group, elevated cerebral regional glucose metabolism was detected in the left hemisphere, including the inferior frontal gyrus, while decreased metabolism was present bilaterally in the right OFC gyrus and hippocampus. Although the authors suggested that differences in level of regional glucose metabolism between cortical and subcortical structures could be central to the pathology of ASP, many of these subjects may not have met diagnostic criteria for ASPD, and, as such, results cannot generalize to the wider group of individuals with rigorously diagnosed ASPD.

A major limitation of the FDG PET studies described above is the narrow representation of ASPD in the study samples. Most studies intentionally recruited BPD participants with IED-R, some of whom happened to have comorbid ASPD. Thus, it is unclear how these results relate to ASPD, since very few studies examined an ASPD diagnosis as a covariate. Furthermore, at least one study whose aim was to report on findings of antisocial individuals did not use structured assessments to diagnose ASPD but instead relied upon self-report psychometric data. FDG PET results of BPD cannot be assumed to translate to ASPD, as some data have shown differing neural correlates between the two disorders.

## PET Studies with Serotonergic Probes

Among the following seven studies that reported the number of ASPD participants scanned, one quarter (67/268) sampled ASPD participants. When serotonergic PERs became available, many researchers started investigating 5-HT transporter (SERT) and 5-HT receptor occupancies in personality disorder populations. The advent of these serotonergic probes provided a new avenue to study the neurochemistry of ASPD.

The radiopharmaceutical [^11^C] McN 5652 was first used to quantify SERT distribution in the brains of 10 individuals with impulsive aggression (2 subjects had comorbid ASPD) and 10 healthy controls [43]. Outcome measures included binding potential (BP_ND_: ratio at equilibrium of specifically bound radioligand to non-displaceable radioligand in tissue) of the SERT and the specific-to-non-specific partition coefficient (V_3_″). The authors reported that both indices of SERT in the ACC were lower in the IED group. Along with the OFC, the ACC provides top-down control of subcortical structures to inhibit impulsive, reactive responding [44]. In addition to the small sample size, another important limitation of this study is that [^11^C] McN 5652 has been linked to high levels of non-specific binding, which limits the ability to measure SERT in regions of low SERT density [45].

Another study [46] that sampled IED-IR utilized the radiopharmaceutical [11C]MDL100907 to quantify 5-HT_2A_R availability in 25 healthy control subjects and two groups of IED-IR and personality-disordered patients: 14 with current physical aggression and 15 without current physical aggression. There were four ASPD subjects in the former group and six ASPD participants in the latter group. When comparing group differences, OFC 5-HT_2A_ BP_p_ (ratio at equilibrium of specifically bound radioligand to amount of total parent radioligand) and BP_ND_ were higher among IED-IR patients with current physical aggression compared to IED-IR subjects without current physical aggression or healthy controls. No other differences were detected between groups or brain regions. Strengths of the study included a medication-free sample, the absence of concurrent major depressive episodes, and no active alcohol or substance abuse. These results imply that increased OFC 5-HT_2A_R availability may be specifically related to IED-IR with current physical aggression.

A notable investigation [47] that measured 5-HT_2A_R density using an alternative radioligand ([^18^F]setoperone) sampled 16 individuals with ASPD with violent behavior and 16 healthy controls. One of the principal findings was decreased dorsolateral prefrontal cortex (DLPFC) 5-HT_2A_R availability among the ASPD group. Reduced DLPFC 5-HT_2A_R density was observed among participants aged 19–24 years compared with healthy controls, while no differences were detected in ASPD subjects aged 25–33 years. Similar to the study by Rosell et al. (2010) [46], an increase in prefrontal cortex (PFC) 5-HT_2A_R availability was present in the participants 34–39 years of age. One criticism of the investigation is that it did not measure aggression with an operationalized construct. On the other hand, this study is one of very few to retain a clinical sample composed entirely of ASPD participants. Thus, these findings should be regarded as especially relevant to the neurochemistry of ASPD with current violence.

Twenty-nine patients with IED-IR that included a substantial contingent of ASPD subjects (*n* = 17) were compared with 30 healthy controls using [^11^C] DASP PET to measure SERT density in the pregenual ACC, amygdala, and other subcortical structures [48]. As noted above, this research group had previously found lower ACC SERT density in the experimental group using a different radiopharmaceutical sensitive to SERT. In the present study, contrary to the authors’ hypothesis, there was no difference in ACC SERT binding between IED-IR participants and healthy subjects. Interestingly, a significant positive correlation between ACC SERT availability and trait callousness, measured using the Dimensional Assessment of Personality Pathology [49], was detected in the IED-IR group. The researchers conjectured that the previously demonstrated association between low presynaptic 5-HT and aggression was mainly applicable to reactive aggression and that proactive aggression, including callousness as one component, could be related to high presynaptic 5-HT. To the authors’ of this review knowledge, this finding has not been replicated in subsequent PET studies.

A dual-tracer PET investigation that sampled males with high impulsive aggression and excluded those with callous-unemotional traits evaluated SERT binding using [^11^C] DASP and 5-HT_2A_R availability with [^11^C]MDL100907 [50••]. As noted, individuals (all male) had no callous-unemotional traits and were grouped into two categories: high impulsivity (HI) and low impulsivity (LI). All HI participants met diagnostic criteria for either ASPD (*n* = 11), BPD (*n* = 7), or both (*n* = 4). Impulsivity was measured using the Psychopathic Personality Inventory-Revised [51] and the Impulsiveness-Venturesome-Empathy Scale [52]. It was not clear how many ASPD participants were actually scanned. However, 22 HI and LI participants completed a [^11^C] DASP scan and 24 HI and LI subjects underwent [^11^C]MDL100907 scanning. The authors reported a group difference among eight brain regions tested in HT_2A_R BP_ND_ between the HI and LI groups, with lower BP_ND_ in the HI group. Conversely, SERT BP_ND_ was significantly higher in the HI group among all of the regions sampled, although results were driven primarily by differences in the brainstem. Among the entire sample, brainstem SERT BP_ND_ was positively correlated with impulsivity, aggression, and ratings of childhood trauma. Furthermore, in the HI group, a positive correlation emerged between brainstem SERT BP_ND_ and a measure of childhood trauma. The authors posited that childhood adverse experiences could have impacted serotonergic gene function to give rise to neurodevelopmental alterations of 5-HT function, ultimately resulting in high impulsivity. Whether these results were due to neurochemical differences present in ASPD or an altered neural substrate underlying high impulsivity could not be determined. Studies involving ASPD participants with varying levels of impulsivity could help parse these results.

A study targeting HT_2A_R binding that used [^18^F] altanserin sampled 33 BPD patients (eight with comorbid ASPD) and 27 healthy controls [53]. The authors reported that BPD participants with comorbid ASPD, compared to those without ASPD, displayed lower BP_ND_ in the basal ganglia, left thalamus, medial temporal cortex, pregenual cingulate, and the ACC. On the other hand, depression, current substance use disorder, and suicide attempter status did not relate to HT_2A_R binding in any brain region. These results suggest that BPD with comorbid ASPD may represent a different phenotype than either disorder alone, with corresponding alterations in HT_2A_R binding.

One novel study to examine serotonergic targets beyond the SERT and HT_2A_R utilized [^11^C]AZ10419360 to measure 5-HT_1B_R BP_ND_ in 19 incarcerated violent offenders (14 individuals with ASPD) and 24 healthy controls [54••]. 5-HT_1B_Rs function as autoreceptors presynaptically and heteroreceptors postsynaptically, and basic science research suggests that 5-HT_1B_ heteroreceptors may mediate aggression and impulsivity [55]. Although no group differences in 5-HT_1B_R BP_ND_ were discerned (OFC, ACC, and striatum were examined), post hoc analyses examined the relationship between the brain regions and clinical characteristics of violent offenders. For example, striatal 5-HT_1B_R BP_ND_ was associated with trait aggression, trait psychopathy, which was primarily related to self-centered impulsivity, and PCL-R score. Trait anger also showed a relationship with ACC and OFC BP_ND_. Moreover, caudate 5-HT_1B_R BP_ND_ exhibited a strong association with trait anger, trait psychopathy, and PCL-R score. Whole-brain voxelwise analysis in the violent offender group replicated the positive correlation of trait anger and caudate/OFC 5-HT_1B_R BP_ND_. Importantly, none of these associations were present in the healthy control group, although it was noted that the low range of trait anger within the comparison group could have been responsible for the group interaction effect. The high proportion of ASPD subjects in this study group helps generalize these findings to the larger population of male ASPD subjects who engage in violent behavior.

Using the same PET scan data as above, the investigators tested whether HT_1B_R BP_ND_ showed a relationship with performance on a computerized version of an emotional Go/NoGo task [56]. The results of the violent offenders and healthy controls were pooled for the analyses. The authors hypothesized that HT_1B_R binding would be positively correlated with the proportion of false alarms in response to threat-related facial expressions (angry and fearful). Primary outcome measures were false alarms for neutral Go trials, where angry and fearful faces functioned as NoGo trials, and false alarms for angry and fearful Go trials, where neutral faces acted as NoGo trials. Post hoc analyses revealed that false alarms in blocks where angry faces were Go stimuli showed a significant positive correlation with ACC HT_1B_R binding. The authors concluded that men (whose level of aggression varied tremendously given that it was a sample of violent offenders and healthy subjects) with high HT_1B_R binding had relative difficulty withholding responses to neutral NoGo stimuli when Go stimuli were angry faces. They suggested that ACC HT_1B_Rs could be implicated in the neural underpinnings of response inhibition when presented with socially threatening cues.

Earlier 5-HT PET studies focused on examining IED-IR subjects with comorbid BPD. In most instances, the proportion of ASPD subjects was minimal. It is, therefore, doubtful whether these findings contribute substantially to our knowledge about the neurochemistry of ASPD. Additionally, some of these studies produced contrary results, as one study reported that ACC SERT binding was lower in IED-IR, while another study, by the same research group, noted that ACC SERT binding was higher. Limitations of the radiopharmaceuticals utilized may have contributed to divergent results. One investigation that studied ASPD exclusively found that lower 5-HT_2A_R density was present in the DLPFC [47]. However, results were only significant in younger populations. An interesting hypothesis advanced by Van de Giessen et al. [48] suggested that increased ACC SERT availability could be related to proactive aggression. However, to date, there is little evidence to support this conclusion. On the other hand, a dual-radiopharmaceutical PET study whose intent was to examine measures of serotonergic function in highly impulsive ASPD groups found that brainstem SERT BP_ND_ was related to both impulsivity and aggression. Finally, more studies of ASPD participants that use radiopharmaceuticals to probe previously uncharted serotonergic receptors, such as the 5-HT_1B_R, will be necessary to advance the field.

## PET Studies with Alternative Probes

In the single study that follows, 18 ASPD participants and 18 controls participated in the PET experiment. To the best of the authors’ knowledge, only one PET study using a non-serotonergic ligand to study ASPD has been published in the literature [21]. [^11^C]Harmine was the radiopharmaceutical employed to measure monoamine oxidase-A (MAO-A) distribution volume (V_T_) in 18 male violent offenders with ASPD and 18 male control participants [21]. MAO-A is a brain enzyme located on outer mitochondrial membranes that degrades 5-HT, dopamine (DA), and norepinephrine [57]. In each study group, nine of the subjects had alcohol dependence. The authors chose the OFC and ventral striatum (VS) as the primary brain regions given their biological abnormalities in ASPD and aggression [58, 59]. A global reduction in MAO-A V_T_ was observed in ASPD, including the a priori regions. Several self-report, clinician-administered, and behavioral measures of impulsivity additionally displayed an inverse correlation with VS V_T_. Results supported previous findings of MAO-A knockout models in rodents exhibiting aggressive behavior [60, 61] and a rare MAO-A missense mutation in human males leading to extreme aggression and impulsivity [62]. The extent to which comorbid alcohol dependence may have altered results is unknown. A subsequent, multi-modal PET-functional magnetic resonance imaging study based on the same sample reported that functional coupling of VS seeds to frontal regions and hippocampus were positively and negatively associated with VS MAO-A V_T_, respectively, and that VS resting state networks were negatively correlated with impulsivity [63]. These results suggest that brain MAO-A phenotypic markers may have relevance to understanding functional connectivity and impulsivity. The disadvantage of this study is that it did not include a healthy control group.

## SPECT and PET Studies of Aggression

A [^99m^Tc] HMPAO SPECT study examined 20 patients with schizophrenia spectrum disorders (SSDs) and a history of severe violence and nine non-violent SSD subjects without a history of violence as a comparison group [64]. Violent patients who had HCR-20 (historical/clinical/risk) [65] scores above 20 showed reduced accrual of [^99m^Tc] HMPAO in left temporal lobes. Further, 12 violent patients manifested bilateral reduced perfusion and 11 subjects had frontally hypoperfused areas. In the control group, only two patients had frontal and temporal lobe hypoperfusion. The results of the violent SSD subjects largely parallel those seen in ASPD as discussed above, which may imply that frontal/temporal hypoperfusion is not as specific a marker of ASPD as it is a more general correlate of aggressive behavior. It is also possible that some of the violent SSD participants had comorbid ASPD that was not measured.

An elegant investigation using FDG PET analyzed brain metabolic activity and physiological measures in participants at rest, during exposure to violent media, and during exposure to emotional, non-violent media [66•]. Study groups were relatively small, albeit carefully designed. Participants included male subjects who had a history of physical fights in the last year and scored above the 75th percentile on the physical aggression scale of the Buss Perry Aggression Questionnaire (BPAQ) [67] (*n* = 12) and another group of males who did not get into fights and scored at or below the 50th percentile on the same aggression questionnaire (*n* = 13). The most robust neuroimaging finding to emerge was hyperactivity of the default mode network (e.g., medial prefrontal cortex, posterior cingulate cortex, precuneus, inferior parietal lobules, and medial temporal regions) in the aggressive versus non-aggressive group as well as hypoactivity of the OFC and cerebellum at rest. These findings were largely replicated during exposure to violent media and suggested a dissection of brain metabolism as a function of trait aggression.

A series of PET studies have examined DA receptor binding in relation to trait aggression. Plavén-Sigray and colleagues [68] used [^11^C]SCH23390, a DA_1_R ligand, to analyze the association between scores on the Verbal Trait Aggression and Physical Trait Aggression scales of the Swedish universities Scales of Personality [69] and DA_1_R availability. Although the striatum was identified as the main target region, the study was highly exploratory and tested numerous extrastriatal areas, presumably because DA_1_Rs are prevalent outside of the striatum [70]. Among the 23 healthy participants (13 males, 10 females) who participated, DA_1_R BP_ND_ in the limbic striatum showed an inverse correlation with physical trait aggression. Since DA_1_R binding was also associated with social desirability, the authors concluded that increased DA_1_R availability was related to high affiliation and low dominance. However, in a follow-up study by the same authors who aimed to replicate this result [71], no relationship emerged between limbic striatum DA_1_R binding and physical trait aggression. Lack of a diverse sample (the second study recruited only males) and relatively small sample sizes may have led to decreased sensitivity to detect associations. The authors also acknowledged that their first result could have occurred as a result of a type I error.

A carefully conceived investigation [72•] that aimed to measure DA release following observation of a violent movie used [^18^F] desmethoxyfallypride ([^18^F]DFMP), a radiotracer that is comparable to raclopride in terms of its binding affinity to DA_2_Rs and DA_3_Rs [73] but has the advantage of slower kinetics and a longer half-life, separated male participants into two groups based on high (*n* = 11) or low (*n* = 12) MAO-A promoter variable nucleotide tandem repeats (VNTRs). The main study objective was to examine the effect of the MAO-A VNTR on stimulus-related DA release and emergence of aggressive behavior. To achieve this aim, subjects were scanned twice under differing conditions: once while watching a violent movie and on another occasion while viewing a neutral movie. Afterwards, the participants completed the PSAP. The two groups did not differ on measures of aggression or psychopathic traits at baseline. Results revealed that the high-activity MAO-A (MAOA-H) group had greater DA release in the ventral caudate nucleus and putamen (calculated as the Δ in DA release while viewing the violent movie compared with the emotionally neutral movie) and displayed increased aggression, according to results from the PSAP, after viewing the violent movie. Similar to many of the other studies reviewed in the manuscript, the sample size was very small. Thus, results should be viewed as tentative. Yet, the data showed that simplified mechanisms linking lower in vitro MAO-A genotypes to greater DA release and subsequent aggression in humans may be less relevant to understanding the influence of the MAO-A VNTR polymorphism on aggression. Moreover, this study supports findings of a previous PET investigation that used [^18^F] FDOPA to investigate the relationship between DA synthesis capacity and aggression, where lower aggression was associated with greater DA storage capacity in striatum and midbrain [74]. One of the main advantages of the study by Schlüter and co-investigators [72•] is that they investigated aspects of both reactive and proactive forms of aggression.

A final [^11^C]SB207145 PET investigation tested the relationship between 5-HT_4_R binding and results from the BPAQ [75]. An inverse relationship between 5-HT_4_R binding and extracellular levels of 5-HT had been previously demonstrated. The authors assembled 18 brain regions to calculate a global volume-weighted 5-HT_4_R BP_ND_ as a biomarker of central 5-HT tone. They also performed post hoc voxel-based analyses. Sixty-one individuals participated in the experiment (47 males and 14 females); analyses were subsequently adjusted for age, sex, 5-HT transporter polymorphism, and type of PET scanner (subjects were scanned between 2006 and 2013 on two different scanners). A positive correlation was discerned between BPAQ total score and global 5-HT_4_R as well as BPAQ physical aggression and global 5-HT_4_R, but only in males. There was no main effect in the mixed-sex sample. Sex interaction effects were present in the association of 5-HT_4_R binding and BPAQ physical aggression. Whole-brain voxel-based analysis, conducted in males who were younger than 50 years old, revealed two clusters where BPAQ total score showed a significant positive correlation with 5-HT_4_R binding: left ACC and left middle cingulate gyrus and right anterior insula and inferior frontal gyrus. The authors concluded that low cerebral levels of 5-HT were correlated with high trait aggression in males but not females. However, the sample may have been underpowered to observe an effect in females and/or there may have been too little variance to detect an association.

## Conclusion

A common theme throughout this paper is the lack of SPECT and PET studies that have examined large samples of ASPD participants or have conducted subgroup analyses of ASPD groups. However, there are some exceptions [14, 15, 19, 21••, 31, 47, 53, 54••]. Most of the reviewed investigations evaluated criminals, psychiatric inmates, and individuals with BPD and IED-R that included some individuals with ASPD. Therefore, it is unclear how relevant these studies are to understanding the neurochemistry of ASPD. Hypoperfusion of frontotemporal regions, alterations in cortical and subcortical 5-HT receptor or transporter function, and reduced MAO-A V_T_ in the OFC and VS are the principal PET and SPECT findings of ASPD. However, results are not uniform, and some studies with similar paradigms yielded conflicting results.

Yet, it is encouraging to note that the use of PET ligands to investigate ASDP has largely paralleled the evolution of radiopharmaceutical development. Still, there are many more molecular targets that could be pursued with existing PERs, and the hope would be that these probes will be utilized to further investigate ASPD. Importantly, some studies clearly differentiated state versus trait aggression in their subjects by scanning them when they were aggressive or non-aggressive. This distinction is important, since neurotransmitter systems likely to be active during aggression may not be responsive when aggression is absent and vice versa.

There is a trend in psychiatric research to investigate basic dimensions of functioning ranging from genomics to neural circuitry, as exemplified by the Research Domain Criteria (RDoC) of the National Institute of Mental Health (https://www.nimh.nih.gov/research-priorities/rdoc/index.shtml; accessed 1/19/09). We have seen this focus in some of the reviewed studies that measured impulsivity, aggression, and psychopathic traits in relation to a molecular target. Ultimately, mapping the symptoms, especially aggression, that characterize ASPD in non-clinical populations may prove to be just as beneficial as studying categorical diagnoses.

In summary, there are relatively few SPECT or PET studies of ASPD. Investigating ASPD and its symptom clusters is highly relevant given the burden that this diagnosis poses to society. To date, there are no pharmacological interventions specifically targeted for ASPD. Examining alternative neuroreceptor systems could be particularly beneficial in this regard to help yield novel pharmacologic treatments.
